# A New Model for Predicting Outbreaks of West Nile Virus

**DOI:** 10.1371/journal.pbio.0040101

**Published:** 2006-02-28

**Authors:** Liza Gross

Infectious diseases were wreaking widespread havoc long before scientists had any idea what caused them. But knowing the pathogenic agents behind today's scourges is just the first step in protecting against deadly outbreaks. Roughly 75% of emerging infectious diseases are zoonotic—humans contract them either directly from infected animals or through vectors that feed on infected animals. West Nile virus is the biggest threat in North America, where *Culex* mosquitoes are the primary vector. Birds are their main target, but mosquitoes also transmit the virus to humans, horses, and other mammals.

Since the virus was first discovered in New York City in 1999, it has infected 20,000 people and killed 770—in stark contrast to the sporadic infections in Europe. The factors behind the North American epidemics are poorly understood, though proposed explanations involve a more virulent strain, North American birds' ineffectual immune response, and a hybrid species of mosquito that prefers humans over birds. In a new study, A. Marm Kilpatrick, Peter Daszak, and their colleagues now present evidence that a shift in *Culex pipiens* mosquito feeding behavior from birds to mammals is also driving the epidemics.

A critical factor in predicting the intensity of a zoonotic epidemic involves determining how the vector's feeding behavior and preferences change over space and time. Birds appear to be West Nile's most competent vertebrate host—they transmit the virus to other mosquitoes, which supports viral reproduction—while humans (and most other mammals) can't transmit the virus. The researchers hypothesized that if mosquitoes bit mostly birds in the summer, then switched to humans in the fall, this behavior could intensify both the summer epidemic in mosquitoes and the subsequent transmission to humans.

To investigate this possibility, Kilpatrick et al. collected data from six sites in Maryland and Washington, D. C., from May through September 2004, to determine the population dynamics of birds and mosquitoes, which taxa *Culex* was targeting, and the epidemiology of the virus. They estimated population densities for mosquitoes and birds at each site, and identified the morphologically cryptic mosquitoes by sequencing their DNA. Over 90% of their catches were *Cx. pipiens*, which were tested for the virus. The researchers determined species of avian and mammalian targets by sequencing the DNA from blood in engorged mosquitoes.

From May to June, the American robin, which represents just 4.5% of the local avian species, accounted for over half of *Cx. pipiens*' meals. As the summer wore on, and robins left their breeding grounds, the probability that humans would provide the blood meal increased 7-fold, while the probability that *Cx. pipiens* would feed on robins declined. Since the birth of new offspring raised the overall numbers of birds during this same period, Kilpatrick et al. concluded that mosquitoes switched to humans when robins—their preferred host—dispersed.

With the data collected from the Washington, D. C., area, the researchers modeled the risk of *Cx. pipiens*–mediated viral transmission to humans based on *Culex* mosquito abundance, the prevalence of *Culex* infection, and the probability that mosquitoes would feed on humans. The model predicted that the risk of human infection peaked in late July to mid-August, declined toward the end of August, then rose slightly at the end of September. The pattern of actual human cases in the area, the authors point out, “showed a strikingly similar pattern.” The model also suggests that the human incidence of West Nile virus would have been much lower if mosquitoes had maintained their June feeding rate throughout the season.

The same pattern was seen in California and Colorado, with a peak abundance of infected *Cx. tarsalis* mosquitoes in June and July, followed by a late-summer spike in human infections. Since mosquitoes feed primarily on birds during early summer, viral load can increase substantially. When mosquitoes switch to humans, the prevalence of infection among mosquitoes increases the chances of a human epidemic. If mosquitoes had fed mostly on humans—wasted meals from the perspective of viral amplification—instead of birds during early summer, prevalence of infection in mosquitoes and then humans would have been greatly reduced.

These feeding shifts appear to be a “continent-wide phenomenon,” the researchers conclude, and may also explain outbreaks of other avian zoonotic viruses. This study highlights the importance of understanding how vector behavior affects transmission of zoonotic pathogens to humans—a crucial step in developing strategies to prevent and control a potential epidemic.

## 

**Figure:**
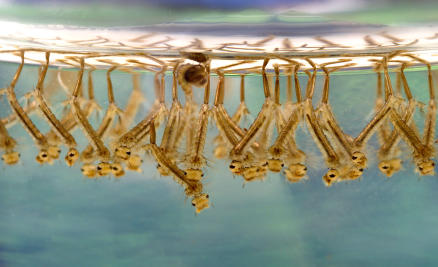
A shift in the feeding behavior of *Culex* mosquitoes (their larvae amass in standing water, as seen above) helps explain the rising incidence of West Nile virus in North America. (Image: James Gathany, CDC)

